# Translational Model to Predict Lung and Prostate Distribution of Levofloxacin in Humans

**DOI:** 10.3390/pharmaceutics18010107

**Published:** 2026-01-13

**Authors:** Estevan Sonego Zimmermann, Teresa Dalla Costa, Brian Cicali, Mohammed Almoslem, Rodrigo Cristofoletti, Stephan Schmidt

**Affiliations:** 1 Center for Pharmacometrics and Systems Pharmacology, Department of Pharmaceutics, College of Pharmacy, University of Florida, Orlando, FL 32827, USA; 2 Pharmacokinetics and PK/PD Modeling Laboratory, Faculty of Pharmacy, Federal University of Rio Grande do Sul, Porto Alegre 90610000, RS, Brazil; 3 Certara, Certara Drug Development Solutions, Randor, PA 19087, USA

**Keywords:** levofloxacin, tissue distribution, PBPK modeling, P-gp, animal-to human extrapolation

## Abstract

**Background/Objectives:** Levofloxacin (LVX) is a fluoroquinolone approved for the treatment of bacterial pneumonia, sinusitis, and prostatitis. Emerging in vitro and preclinical evidence suggests that efflux transporters are involved in LVX’s target tissue site distribution. **Methods**: The objective of this research was to characterize tissue exposure using a physiologically based pharmacokinetic (PBPK) model to be able to make more educated choices for optimal doses using target site pharmacokinetics data. **Results**: The final PBPK model in humans was applied to simulate free target site concentrations of LVX in lung and prostate, linking to minimum inhibitory concentrations (MIC) to assess appropriateness of currently approved dosing regimens for infections in both tissues. The clinical PBPK model was able to reproduce total plasma as well as free lung and prostate exposure of LVX in humans. Efflux transporters participate in LVX distribution to prostatic but not pulmonary tissue. Our results show a good penetration of LVX in both tissues with unbound partition coefficient (Kp,uu) equal to 0.79 and 0.72 for lung and prostate, respectively. Since LVX penetration in lung and prostate is similar, different sensitivities of the pathogens to LVX will dictate the effectiveness of the approved therapeutic regimen in the treatment of bacterial pneumonia, sinusitis, and prostatitis. **Conclusions**: Our research provides relevant insight into LVX’s target site exposure in lung and prostate. When integrated with pathogen-specific susceptibility data, these findings can be applied to refine current dosing regimens and help optimize the pharmacological treatment outcomes.

## 1. Introduction

The continuous development and spread of antimicrobial resistance in conjunction with the lack of novel antibiotics represents a serious challenge for human society [1,2]. To address this challenge, 16 novel antibiotics were approved by the U.S. Food and Drug Administration (FDA) and the European Medicines Agency (EMEA) between 2017 and 2023. However, only two of them were developed to target problem pathogens according to the World Health Organization [3]. The optimal use of currently existing antibiotics, either alone or in combination, is consequently becoming even more important to have safe and effective treatment options available to patients and to minimize the emergence of bacterial resistance.

Optimal treatment and dosing regimens are routinely based on total concentrations in blood or plasma in conjunction with bacterial susceptibility. However, total concentrations in blood or plasma may or may not be accurately reflective of free, pharmacologically active concentrations in the extracellular space fluid of tissue (e.g., lung, liver, heart, kidney, brain, or testis), where most pathogens reside [4]. Therefore, it is important to determine free, unbound target tissue concentrations and how they relate to total concentrations in blood or plasma. The availability of drug at the target tissue site can depend on the involvement of transporters [5,6]. P-glycoprotein (P-gp) is the most clinically important efflux transporter. It is a member of the ABC family and is primarily expressed in the apical (AP) membrane of biological barriers [7,8]. The involvement of P-gp may result in a significant tissue access barrier [9] and thus insufficient pharmacologically active target tissue site concentrations.

Levofloxacin (LVX) is a fluoroquinolone approved for the treatment of bacterial pneumonia, sinusitis, prostatitis, and several other infections. LVX is mainly eliminated renally through glomerular filtration and tubular secretion. Approximately 80% of the drug is recovered unchanged in urine, and the metabolism is considered minimal [10]. After oral administration, it exhibits dose-proportional pharmacokinetics across the dose range of 50–1000 mg [11]. LVX readily distributes into various tissues. Tissue biopsy data suggest that total LVX concentrations in prostate [12,13] and lung [14] exceed those in plasma. However, concentrations derived from tissue homogenates represent a mixture of bound and unbound intracellular as well as extracellular concentrations. Tissue homogenate data may consequently paint a biased picture of unbound concentrations in the interstitial space fluid (ISF) of target tissues [15]. Evidence further suggests that efflux transporters are involved in LVX’s tissue distribution [16,17,18].

Microdialysis is a semi-invasive sampling technique that allows for the assessment of unbound ISF concentrations of virtually any tissue over time [19,20]. It has been successfully used to improve our knowledge of tissue distribution, including the impact of transporters on unbound tissue concentrations [21]. Integration of this knowledge in a pharmacokinetic/pharmacodynamic (PK/PD) context will further enhance our understanding of LVX’s exposure–response relationships and inform optimal dosing and treatment decisions. There are relevant studies successfully applying microdialysis to investigate LVX’s tissue distribution and assess the role of P-gp in prostate [22] and lung in absence and presence of P-gp inhibitor [23]. These studies now serve as the experimental basis for the development of a physiologically based pharmacokinetic (PBPK) model in rats, which once developed and verified, will be expanded to humans by accounting for physiological differences between species. Ultimately, this PBPK model will be used in simulations to link free, unbound concentrations in lung and prostate to minimum inhibitory concentrations (MIC) to assess the appropriateness of currently approved dosing regimens for infections in these tissues.

## 2. Materials and Methods

### 2.1. Software

GastroPlus^™^ version 9.8 (Simulations Plus, Inc., Lancaster, CA, USA) was used to develop the LVX PBPK models in both rats and humans. Default anatomical and physiological parameters for rats and humans were used for model development. Kp values were manually optimized by fitting (details see Section 2.3). The Population Estimates for Age Related (PEAR) Physiology module was used to generate virtual populations. Data from published preclinical and clinical trials were extracted using Graph Grabber v.2.0.2 (Quintessa Ltd., Warrington, UK). Goodness-of-fit plots comparing simulated and observed plasma/tissue concentrations were performed in R (version 4.1.2, R Foundation for statistical Computing) and RStudio (version 2024.12.1-563, Posit, Inc., Boston, MA, USA). Graphical abstract was created in BioRender (Toronto, ON, Canada, https://www.biorender.com).

### 2.2. Model Development Overview

**Figure 1:**
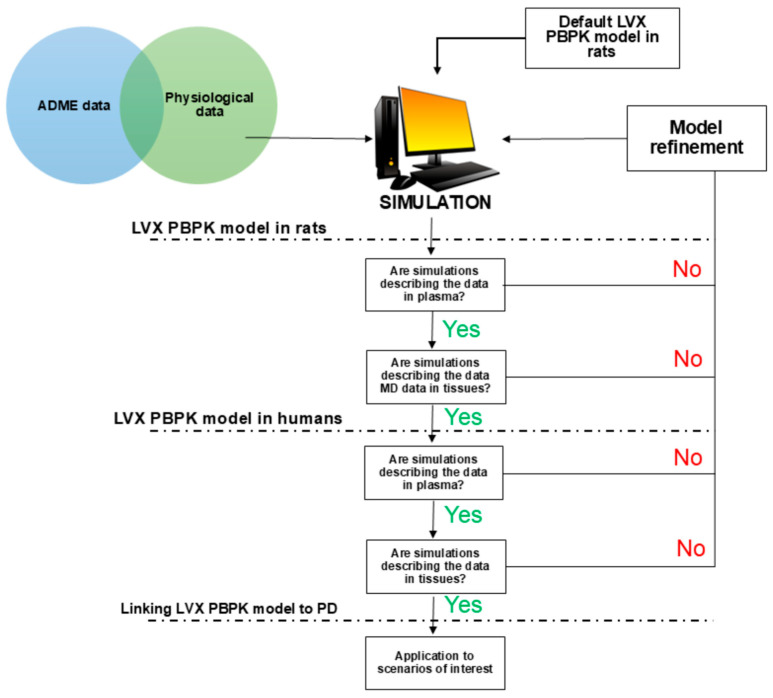
Overview of workflow for PBPK model development and verification.

Figure 1 provides an overview of the stepwise modeling workflow. First, we developed the rat PBPK model using experimental LVX microdialysis data in the absence of TAR [23] to validate the model. Once developed and verified in rats, the PBPK model was scaled to humans by accounting for physiological differences, such as tissue composition and organ blood flow between rats and humans. Following external verification in humans using available literature data, the model was used to predict unbound LVX concentrations in the ISF of human lung and prostate, which were compared in a final step to the MIC of infecting organisms to assess the likelihood of therapeutic success.

### 2.3. Development and Verification of the LVX PBPK in Rats

Drug-specific parameters for LVX were obtained from the literature and are shown in Table 1. LVX renal clearance was parametrized as fu*GFR, where *fu* represents the fraction unbound in plasma and GFR the glomerular filtration rate. Drug disposition was assumed to be perfusion-limited for all tissues including lung and prostate. As prostatic tissue is not available by default in GastroPlus^™^, the volume of the reproductive organ was adjusted to physiological prostatic volume without changes in blood flow and tissue composition. Tissue physiology is a relevant factor that might impact drug distribution into tissues. Following intravenous administration, drug must first penetrate several cell layers to reach the ISF of tissues. Drug needs to initially diffuse through capillary endothelial and vascular basement membranes of the prostate to reach the ISF, from where it must cross a basal cell layer located between the basement membrane and the luminal cells (double layer stratified). The presence of gap junctional proteins between basal and luminal cells provides an extra diffusion barrier [24]. In comparison, LVX needs to cross the alveolar capillary barrier to reach its site of action in the lung. This barrier is composed of capillary lumen, connective tissue, and epithelium. The latter is particularly relevant for target distribution because it is less permeable than the other layers due to the presence of zonula occludens between cells [25,26].

The in silico methods available in GastroPlus^™^ were tested to estimate tissue-to-plasma partition coefficients (Kp) and volume of distribution at steady-state (Vss). The best method (Lukacova with lysosomes) was chosen based on a comparative analysis of Vss values (predicted versus observation) in conjunction with visual inspection of goodness of fit plots. Since the prostate is not a default organ in GastroPlus^™^, the reproductive organ was modified to integrate the prostatic tissue. Briefly, organ volume was modified to emulate prostate tissue volume as 0.65 mL while blood flow was rebalancing, and tissue composition values were set for reproductive organs. As the preclinical PK data was obtained from anesthetized rats, an important additional step was to account for the effect of anesthesia on organ blood flow. Evidence from the literature suggests that cardiac output decreases to 4.4 L/h/Kg or 0.367 mL/s [27] in rats under anesthesia and was adjusted in GastroPlus^™^ accordingly. Kp lung was predicted by Lukacova with lysosomes method without any optimization. To account for P-gp in prostate, Kp was optimized using the control arm (without TAR) of the microdialysis study. S + 9.5 method was used to assess the fraction unbound in tissue (fut) as well as unbound tissue concentrations for both organs.

The predictive performance of the rat PBPK model was assessed by comparing model predictions for unbound ISF concentrations of lung and prostate as well as total concentration in blood/plasma to available experimental data [22,23]. Model predictions were deemed appropriate if PK parameters derived from simulations were contained within a 2-fold range of those derived from observations [28,29]. Visual predictive checks were performed to assess the appropriateness of respective population variance estimates. To that end, a virtual population consisting of 100 male rats was used [22,23].

### 2.4. Development and Verification of the Human LVX PBPK Model

Once developed and verified, the rat PBPK model for LVX was scaled to humans by accounting for physiological differences between rats and humans (Table 1). The scaled renal clearance of 5.18 L/h accounted for 80% of LVX’s total clearance in humans. The remaining 20% (1.3 L/h) was attributed to hepatic clearance, which is in line with the literature [11]. The volume for human prostate (21 mL) was obtained from the literature [30,31]. Kp value for lung was predicted using Lukacova with lysosomes method without any optimization. Kp prostate was assumed to be the same for rats and humans based on experimental data (Table 1). The unbound partition coefficients (Kp,uu) were calculated by *ƒ*AUC_free tissue 0-inf_ over *ƒ*AUC_free plasma 0-inf_ ratio for last dose after multiple dosing regimen.

**Table 1:** Key physicochemical and biopharmaceutical parameters for LVX.

Parameter	Values	REF
Molecular weight (g/mol)	361.4	-
Log D at pH 7.4	−1.35 (pH 7.0)	[32]
pKa1 and pKa2	5.7 and 7.9	[33]
Plasma protein binding (ƒup)	55% (rats) 70% (humans)	[22] [11,34]
Reference Solubility at pH 6.7	272 mg/L	[10]
Diffusion coefficient	0.75 × 10^−5^ cm/s^2^	ADMET 10
Blood:plasma concentration ratio (Rbp)	0.9 (rats)	ADMET 10
	1.0 (Humans)	[35]
Clearance		
Renal Clearance	0.160 L/h (Rats)	Estimated ƒu*GFR
	5.179 L/h (Humans)	Estimated ƒu*GFR
Metabolic Clearance	1.300 L/h (Humans)	Calculated
Partition coefficient (Kp)		
Kp Lung	4.84 (rats)/4.38 (humans)	Lukacova with lysosomes
Kp Prostate	5.0	Optimized
ƒut calculation method	S + 9.5v.	Default
ƒut Lung	0.100 (rats)/0.141 (humans)	Calculated
ƒut Prostate	0.080 (rats)/0.123 (humans)	Calculated

ƒut = fraction unbound in tissue.

The predictive performance of the human PBPK model was assessed by recapitulating available clinical data on LVX concentrations in plasma (total LVX), lung (free LVX), and prostate (total LVX) [12,36,37,38]. The model was deemed appropriate if simulated PK parameters were within a 2-fold range in comparison to the respective observed data [28,29].

### 2.5. Establishment of a PBPK/PD Model to Predict Clinical Efficacy

Once developed and verified, the human LVX PBPK model was linked to MIC to evaluate the adequacy of approved dosing regimens against clinically relevant pathogens, including *Pseudomonas aeruginosa*, *Staphylococcus pneumoniae*, *Staphylococcus aureus*, *Haemophilus influenzae*, and *Enterobacterales*. The free area under the concentration-time curve over MIC ratio (*ƒ*AUC/MIC) ≥ 30 is considered the most appropriate PK/PD indices for fluoroquinolones [39,40,41,42]. This index was used as the target to evaluate the antimicrobial efficacy of LVX in lung and prostate following four different dosing regimens: prostatitis (500 mg every 24 h for 28 days); respiratory tract infections (500 or 750 mg every 24 h for 5, 7 or 14 days [10]) as well as two hypothetical scenarios (1000 and 1250 mg) to assess the potential benefit of increased exposure. *ƒ*AUC was calculated using the trapezoidal rule and *ƒ*AUC_0–24 h_/MIC indices were calculated for pathogens specified above. MIC ranges were obtained from Laboratory Standards Institute [43]. Within MIC ranges, the highest susceptible MIC value is particularly relevant for clinical practice and was therefore selected to calculate the *ƒ*AUC/MIC ratios. Since LVX’s half-life is ~6–8 h, steady-state free AUC, i.e., AUC_free,tau,SS_ was used to calculate *ƒ*AUC/MIC ratios.

## 3. Results

### 3.1. Rat PBPK Model

**Figure 2:**
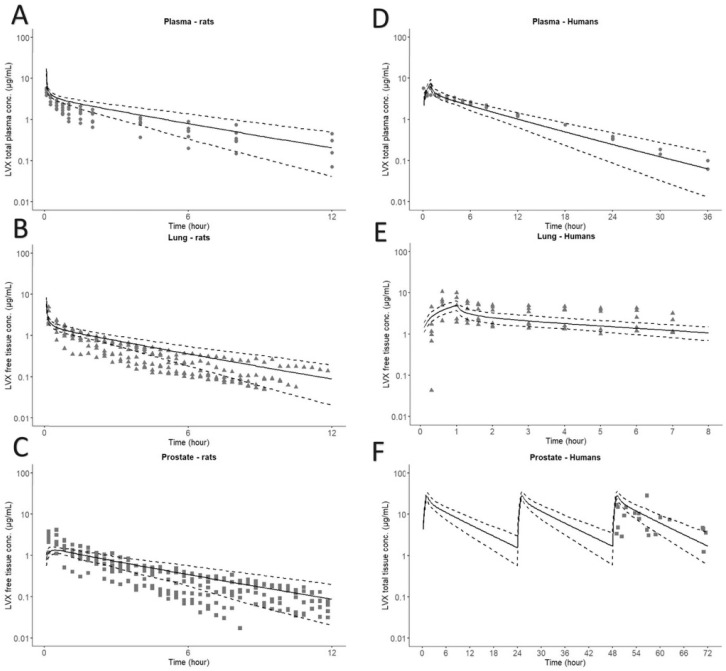
Model verification for rat (**A**–**C**) and human (**D**–**F**) PBPK models. Comparison of simulated (lines) and observed plasma (dots), lung (triangles) and prostate (squares) concentration–time profiles of LVX after 7 mg/Kg i.v. bolus dose and 500 mg 1 h infusion for rats (*n* = 6–7/group) and humans (*n* = 6–22/group), respectively. Solid black and dashed lines represent mean and 5–95th per-centiles of population simulation (*n* = 100 subjects), respectively.

Of all distribution algorithms tested, the Lukacova with lysosomes method provided the best fit. The estimated Vss of 0.66 L (2.2 L/Kg) was similar to the experimentally determined value of 3.2 L/kg [23] and 4.0 L/kg [22]. The final model parameters for the rat and the human PBPK models are summarized in Table 1. Our model was able to capture total LVX concentrations in plasma as well as free concentrations in the lung and prostate reasonably well as shown in Figure 2. Population Predictions (PRED) vs. observed (OBS) ratios were contained within the two-fold acceptance criteria (Table 2).

### 3.2. Human PBPK Model

The clinical PBPK model successfully recapitulate the total LVX concentrations in plasma and prostate as well as free concentrations in the lung, demonstrating good concordance with central tendency of the observations in plasma from three different clinical studies after administrations of LVX 500 mg as 1 h infusion (Figure 2) [11,36,37]. PRED/OBS ratios between PK metrics were within the acceptance criteria (Table 3). The estimated Vss of 115.2 L (1.65 L/kg) was very similar to literature-reported values (117 L [37]; 105 L [36]; 89–112 L [11]).

### 3.3. Model Application to Predict LVX Efficacy Against Pulmonary and Prostate Infections in Humans

The results of our simulations indicate that LVX 500 mg every 24 h generates unbound AUC_0-inf_ (last dose) as 35.16, 27.74 and 25.15 µg.h/mL for plasma, lung and prostate, respectively. Thereby, the Kp,uu calculated for lung (Kp,uu: 0.79) and prostate (Kp,uu: 0.72) suggest that LVX penetrates well into both tissues. Our results further suggest that 500 mg of LVX daily provide high enough unbound concentrations in the lung to effectively treat infections caused by *Enterobacteriaceae*. However, a higher dose of 750 mg is needed to effectively treat *P. aeruginosa* and *S. aureus* infections in the lung. Even higher doses of 1250 mg are needed to achieve sufficient coverage for *S. pneumoniae* and *H. influenza* in the lung (Table 4). In comparison, 500 mg QD is sufficient to effectively treat infections caused by susceptible *Enterobacteriaceae* in the prostate, while a dose of 750 mg dose is needed for *P. aeruginosa* and *S. aureus*. All the investigated doses failed to produce high enough unbound exposures to effectively treat *S. pneumoniae* and *H. influenza* infections in the prostate (Table 5).

## 4. Discussion

We successfully developed a translational PBPK model for LVX by integrating available preclinical data in rats and clinical data in humans in a stepwise fashion. For rats, our analysis shows that P-gp does not impact distribution into the lung (perfusion-limited distribution), whereas P-gp plays a significant role for LVX’s distribution into the prostate following systemic administration. These findings are supported by the fact that LVX is rapidly distributed through the tissues and mainly mediated by passive diffusion. A previous study investigated the LVX tissue distribution using microdialysis in rat muscle and lung after intravenous infusion under steady-state conditions. The results for AUC_free tissue_/AUC_free plasma_ ratios are 1.0 and 1.1 for muscle and lung, respectively [44]. Although P-gp expression levels are significant in lungs, P-gp is mainly expressed at the apical side of bronchial epithelium, i.e., at the lung/air interface [45,46], which renders it uninfluential for distribution from blood/plasma into the lung, whereas the co-administration of tariquidar, a strong P-gp inhibitor [23], significantly increased distribution into the prostate. Initially, permeability-limited model was tested to describe the P-gp efflux transport for prostatic tissue which requires the input of V_max_ (maximum transporter velocity) and K_m_ (Michaelis constant). There are few studies describing P-gp efflux transport for LVX as well as reporting K_m_ values determined by Caco-2 [17] and LLC-PK1 cells [18,47]. However, these cell systems are derived from human colon carcinoma and pig kidney cortex, respectively, which are not representative of the prostatic barrier. Estimating K_m_ from in vivo data was another option considered, but an accurate estimation of this parameter requires different dose levels above and below K_m_ which are currently unavailable. Therefore, perfusion-limited was chosen as more viable alternative to describe the tissue distribution in prostate.

There is no literature evidence suggesting that there is a significant between-species difference in tissue distribution for LVX. Therefore, Kp values for lung were predicted by Lukacova with the lysosomes method and prostate kept identical for rats and humans, followed by a verification step using clinical data validating this modeling assumption. A similar rational has been described in the literature as alternatives to predict tissue distribution in humans using data from rodents [48,49]. A microdialysis study in human found AUC_free tissue_/AUC_free plasma_ (AUC_0–10 h_) ratio equal to 1.1 in subcutaneous adipose tissues in healthy subjects [50]. A similar study in healthy volunteers reported AUC_free tissue_/AUC_free plasma_ (AUC_0-inf_) ratios of 1.1 and 0.9 for subcutaneous adipose tissue and skeletal muscle, respectively [51]. Similar to rodents, the ratios close to unity in humans characterize the passive diffusion as the major distribution mechanism of LVX. It provides scientific elements to support the choice of perfusion-limited model for all organs. To confirm the accuracy of the transitional modeling strategy, the model verification step was performed using clinical data from biopsy in prostate [12] and microdialysis in lung [38]. The good agreement between model prediction and clinical data for both tissues indicates that the PBPK model is able to simulate the unbound concentration in different clinical scenarios.

Once developed and verified, the translational PBPK model was applied to evaluate the adequacy of currently approved LVX dosing regimens for treating lung and prostate infections with clinically relevant organisms. Our simulation results suggest that LVX exposure in lung tissue after 750 mg would be efficacious against infection caused by *Enterobacteriaceae*, *P. aeruginosa*, and *S. aureus*. Higher doses would be needed to adequately treat respiratory infections caused by *S. pneumoniae* and *H. influenza* (Table 4). This is partially in agreement with the current dose regimens approved for LVX. The maximum daily dose recommended is 750 mg for patients with nosocomial and community-acquired pneumonia (normal renal function). It is important to highlight that current dose recommendations do not cover all the pathogens and specific clinically relevant scenarios should be carefully considered case by case. The need for potentially higher doses for treating infections caused by these pathogens was realized by Conte Jr and collaborators [52], who evaluated the benefit of a 1000 mg daily dose given intravenously. Plasma, bronchoalveolar fluid, and alveolar cells were matrixes quantified from health volunteers. Based on C_max_/MIC_90_ and AUC/MIC_90_ ratios, the authors emphasize that 1000 mg represents advantages in terms of PK characteristics for the treatment of community-acquired respiratory pneumonia. Nevertheless, doses higher than 750 mg might increase the potential risk of side effects to the patient. Recently, relevant adverse effects have been reported for fluoroquinolones including tendon ruptures, aortic aneurysms, low blood sugar, and mental health such as disorientation, agitation, nervousness, memory impairment, and delirium [10,53,54]. Despite the low incidence, the potential risk/benefit to the patient should be considered carefully for each situation.

The standard dose for treating chronic bacterial prostatitis is 500 mg daily for 28 days [10]. Clinical outcomes indicate that a 750 mg dose for 21 days can achieve a success rate comparable to the standard treatment [55]. Microorganisms often associated with chronic bacterial prostatitis include *Enterobacteriaceae*, *P. aeruginosa*, and *S. aureus* [56,57], and our results are in accordance with literature reports indicating that 500 mg and 750 mg doses are appropriate to eradicate *Enterobacteriaceae* and *P. aeruginosa*/*S. aureus*, respectively. Nonetheless, the pharmacological treatment of bacterial prostatitis remains a challenge and associated with high recurrence rates. As described previously, the penetration of fluoroquinolones can decrease more than 70% in chronic infection [58] and the current PBPK model does not account for the physiological changes during infected conditions. Since quantitative models serve as a knowledge repository for the data generated in drug product development programs, emergence of new data related to LVX will guide further refinement of the current LVX PBPK model and, the strategic application and exploitation of the knowledge contained in such a model can lead to more efficient and cost-effective treatment.

PK/PD indices have made valuable scientific contribution to enhancing the development of fluoroquinolones and optimizing dosing regimens across various clinically relevant scenarios [59,60,61,62]. Nonetheless, this approach often relies on blood/plasma concentrations, while corresponding target site PK/PD indices still unavailable. Despite the well-established relevance of incorporating unbound drug concentrations into PK/PD indices [14], existing indices do not yet accurately capture PK/PD dynamics at the tissue target site. Accordingly, further investigation is warranted to bridge this critical knowledge gap. Another limitation of the present research is that we did not account for variability around MIC values due to pathogen resistance mechanisms, and instead assumed constant values based on CLSI reference [43]. It is important to highlight that the present PBPK model was built under the assumption that LVX tissue penetration is not significantly affected by infections. This assumption is based on the preclinical study that found no difference between LVX exposure in healthy and inflamed adipose tissue [50]. However, a recent publication demonstrated in rodents that ciprofloxacin penetration decreased significantly in infected lung. The tissue penetration factors in lung interstitium were 1.69 (healthy), 1.02 (acute infection), and 0.44 (chronic infection), which represents a reduction of 40% and 74% for acute and chronic conditions, respectively. These findings suggest that disease progression is also a critical factor for tissue distribution of fluoroquinolones [58]. As drug penetration is significantly lower for infected tissues, the dose necessary to achieve the free target concentration might be higher, particularly in chronic conditions. It is important to acknowledge that transporters other than P-gp may participate in LVX distribution. Findings from a recent microdialysis study in rats suggest that multidrug resistance-associated proteins (MRPs) function as relevant efflux transporters modulating LVX penetration into the brain [63]. In the future, additional transporters can be integrated into the current PBPK model and contribute to a more comprehensive understanding of LVX tissue distribution.

## 5. Conclusions

In summary, we developed a preclinical (rat) PBPK model to characterize LVX’s tissue distribution in lung and prostate. Our model accounts for the anesthesia effect on cardiac output and able to recapitulate the tissue distribution in lung and prostate. Once developed and qualified in rats, we successfully scaled the PBPK model to humans by accounting for differences in plasma protein binding and clearance, among other physiological parameters. This clinical PBPK model was verified using clinical data in plasma (total concentration), biopsy for prostate (total concentration), and microdialysis for lung (unbound concentration). The final clinical PBPK model was applied to predict the free concentrations of LVX in lung and prostate in conjunction with PD information (MIC) to assess the effectiveness of approved therapeutic dose regimens for LVX against different pathogens. Our research provides relevant insight into LVX’s target site exposure in lung and prostate. When integrated with pathogen-specific susceptibility data, these findings can be applied to refine current dosing regimens and help optimize the pharmacological treatment outcomes.

## Figures and Tables

**Table 2:** Comparative analysis between observation vs. simulation free AUC in lung and prostate using the rat PBPK model.

	Observed	Simulated	90% CI	Ratio (Pred/Obs)
AUC _plasma total 0-t_ (µg.h/mL)	10.58	11.68	11.25–12.12	1.10
AUC _plasma total 0-inf_ (µg.h/mL)	12.18	13.10	12.49–13.72	1.08
AUC _lung free 0-t_ (µg.h/mL)	5.49	6.08	5.91–6.26	1.11
AUC _lung free 0-inf_ (µg.h/mL)	5.78	6.60	6.38–6.83	1.14
AUC _prostate free 0-t_ (µg.h/mL)	4.68	5.56	5.39–5.74	1.19
AUC _prostate free 0-t_ (µg.h/mL)	5.39	6.05	5.82–6.29	1.12

Data are shown as mean. CI = Confidence interval.

**Table 3:** Comparative analysis between observation vs. simulation total concentration for plasma and prostate and free concentration for lung using the human PBPK model.

	Observation	Simulation	90% CI	Ratio (Pred/Obs)
AUC _plasma total 0-t_ (µg.h/mL)	35.74	39.54	38.46–40.62	1.11
AUC _plasma total 0-inf_ (µg.h/mL)	36.58	40.08	38.93–41.23	1.10
AUC _lung free 0-t_ (µg.h/mL)	20.38	14.90	14.52–15.27	0.73
AUC _lung free 0-inf_ (µg.h/mL)	36.34	23.35	22.69–24.01	0.65
AUC _prostate total 0-t_ (µg.h/mL)	160.96	185.89	179.75–192.02	1.15
AUC _prostate total 0-inf_ (µg.h/mL)	211.61	202.88	195.14–210.65	0.96

Data are shown as mean. CI = Confidence interval.

**Table 4:** Pharmacokinetic/pharmacodynamic (PK/PD) indices associated with the simulated approved dosage regimens for levofloxacin (LVX) in lung using final PBPK model in humans. Red flag (AUC_free_/MIC < 30) and green flag (AUC_free_/MIC > 30).

Pathogen	CLSI ^1^ Susceptible (mg/L)	Lung AUC_free_/MIC			
		500 mg/24 h AUC_free 96–120 h_ 25.9 μg.h/mL	750 mg/24 h AUC_free 96–120 h_ 38.3 μg.h/mL	1000 mg/24 h AUC_free 96–120 h_ 49.5 μg.h/mL	1250 mg/24 h AUC_free 96–120 h_ 62.6 μg.h/mL
*P. aeruginosa*	≤1	25.9	38.3	49.5	62.6
*S. pneumoniae*	≤2	13.0	19.2	24.8	31.3
*S. aureus*	≤1	25.9	38.3	49.5	62.6
*H. influenza*	≤2	13.0	19.2	24.8	31.3
*Enterobacteriaceae*	≤0.5	51.8	76.6	NC	NC

^1^ CLSI—Clinical and laboratory standard institute—documents M100, 29th ed.

**Table 5:** Pharmacokinetic/pharmacodynamic (PK/PD) indices associated with the simulated approved dosage regimens for levofloxacin (LVX) in prostate using final PBPK model in humans. Red flag (AUC_free_/MIC < 30) and green flag (AUC_free_/MIC > 30).

Pathogen	CLSI ^1^ Susceptible (mg/L)	Prostate AUC_free_/MIC			
		500 mg/24 h AUC_free 96–120 h_ 23.4 μg.h/mL	750 mg/24 h AUC_free 96–120 h_ 35.4 μg.h/mL	1000 mg/24 h AUC_free 96–120 h_ 45.7 μg.h/mL	1250 mg/24 h AUC_free 96–120 h_ 58.0 μg.h/mL
*P. aeruginosa*	≤1	23.4	35.4	45.7	58.0
*S. pneumoniae*	≤2	11.7	17.7	22.9	29.0
*S. aureus*	≤1	23.4	35.4	45.7	58.0
*H. influenza*	≤2	11.7	17.7	22.9	29.0
*Enterobacteriaceae*	≤0.5	46.8	70.8	NC	NC

^1^ CLSI—Clinical and laboratory standard institute—documents M100, 29th ed.

## Data Availability

Data will be made available upon request.

## References

[1] Tacconelli E., Carrara E., Savoldi A., Harbarth S., Mendelson M., Monnet D.L., Pulcini C., Kahlmeter G., Kluytmans J., Carmeli Y. (2018). WHO Pathogens Priority List Working Group. Discovery, research, and development of new antibiotics: The WHO priority list of antibiotic-resistant bacteria and tuberculosis. Lancet Infect. Dis..

[2] European Centre for Disease Prevention and Control, European Medicines Agency (2009). A Call to Narrow the Gap Between Multidrug-Resistant Bacteria in the EU and Development of New Antibacterial Agents.

[3] World Health Organization (2024). 2023 Antibacterial Agents in Clinical and Preclinical Development: An Overview and Analysis.

[4] Liu P., Müller M., Derendorf H. (2002). Rational dosing of antibiotics: The use of plasma concentrations versus tissue concentrations. Int. J. Antimicrob. Agents..

[5] Theuretzbacher U. (2007). Tissue penetration of antibacterial agents: How should this be incorporated into pharmacodynamic analyses?. Curr. Opin. Pharmacol..

[6] Gonzalez D., Schmidt S., Derendorf H. (2013). Importance of relating efficacy measures to unbound drug concentrations for anti-infective agents. Clin. Microbiol. Rev..

[7] Schinkel A.H., Jonker J.W. (2003). Mammalian drug efflux transporters of the ATP binding cassette (ABC) family: An overview. Adv. Drug Deliv. Rev..

[8] Staud F., Ceckova M., Micuda S., Pavek P. (2010). Expression and function of P-glycoprotein in normal tissues: Effect on pharmacokinetics. Methods Mol. Biol..

[9] Leslie E.M., Deeley R.G., Coleb S.P. (2005). Multidrug resistance proteins: Role of P-glycoprotein, MRP1, MRP2, and BCRP (ABCG2) in tissue defense. Toxicol. Appl. Pharmacol..

[10] Janssen Pharmaceutica U.S. LLC (2008). LEVAQUIN^®^ (Levofloxacin) [Package Insert and Label Information].

[11] Fish D.N., Chow A.T. (1997). The clinical pharmacokinetics of levofloxacin. Clin. Pharmacokinet..

[12] Drusano G.L., Preston S.L., Van Guilder M., North D., Gombert M., Oefelein M., Boccumini L., Weisinger B., Corrado M., Kahn J. (2000). A population pharmacokinetic analysis of the penetration of the prostate by levofloxacin. Antimicrob. Agents Chemother..

[13] Bulitta J.B., Kinzig M., Naber C.K., Wagenlehner F.M., Sauber C., Landersdorfer C.B., Soergel F., Naber K.G. (2011). Population pharmacokinetics and penetration into prostatic, seminal, and vaginal fluid for ciprofloxacin, levofloxacin, and their combination. Chemotherapy.

[14] Lee L.J., Sha X., Gotfried M.H., Howard J.R., Dix R.K., Fish D.N. (1998). Penetration of levofloxacin into lung tissue after oral administration to subjects undergoing lung biopsy or lobectomy. Pharmacotherapy.

[15] Mouton J.W., Theuretzbacher U., Craig W.A., Tulkens P.M., Derendorf H., Cars O. (2008). Tissue concentrations: Do we ever learn?. J. Antimicrob. Chemother..

[16] Brillault J., De Castro W.V., Couet W. (2010). Relative contributions of active mediated transport and passive diffusion of fluoroquinolones with various lipophilicities in a Calu-3 lung epithelial cell model. Antimicrob. Agents Chemother..

[17] Yamaguchi H., Yano I., Hashimoto Y., Inui K.I. (2000). Secretory mechanisms of grepafloxacin and levofloxacin in the human intestinal cell line caco-2. J. Pharmacol. Exp. Ther..

[18] Ito T., Yano I., Tanaka K., Inui K.I. (1997). Transport of quinolone antibacterial drugs by human P-glycoprotein expressed in a kidney epithelial cell line, LLC-PK1. J. Pharmacol. Exp. Ther..

[19] Brunner M., Derendorf H., Müller M. (2005). Microdialysis for in vivo pharmacokinetic/pharmacodynamic characterization of anti-infective drugs. Curr. Opin. Pharmacol..

[20] Chaurasia C.S., Müller M., Bashaw E.D., Benfeldt E., Bolinder J., Bullock R., Bungay P.M., DeLange E.C., Derendorf H., Elmquist W.F. (2007). AAPS-FDA workshop white paper: Microdialysis principles, application and regulatory perspectives. Pharm. Res..

[21] Hammarlund-Udenaes M. (2017). Microdialysis as an Important Technique in Systems Pharmacology—A Historical and Methodological Review. AAPS J..

[22] Hurtado F.K., Weber B., Derendorf H., Hochhaus G., Dalla Costa T. (2014). Population pharmacokinetic modeling of the unbound levofloxacin concentrations in rat plasma and prostate tissue measured by microdialysis. Antimicrob. Agents Chemother..

[23] Zimmermann E.S., Laureano J.V., Dos Santos C.N., Schmidt S., Lagishetty C.V., de Castro W.V., Dalla Costa T. (2016). Simultaneous Semi-mechanistic Population Analyses of Levofloxacin in Plasma, Lung, and Prostate to Describe the Influence of Efflux Transporters on Drug Distribution following Intravenous and Intratracheal Administration. Antimicrob. Agents Chemother..

[24] El-Alfy M., Pelletier G., Hermo L.S., Labrie F. (2000). Unique features of the basal cells of human prostate epithelium. Microsc. Res. Tech..

[25] Knudsen L., Ochs M. (2018). The micromechanics of lung alveoli: Structure and function of surfactant and tissue components. Histochem. Cell Biol..

[26] Wenzler E., Fraidenburg D.R., Scardina T., Danziger L.H. (2016). Inhaled Antibiotics for Gram-Negative Respiratory Infections. Clin. Microbiol. Rev..

[27] Sweet C.S., Emmert S.E., Seymour A.A., Stabilito I.I., Oppenheimer L. (1987). Measurement of cardiac output in anesthetized rats by dye dilution using a fiberoptic catheter. J. Pharmacol. Methods.

[28] Jones H.M., Chen Y., Gibson C., Heimbach T., Parrott N., Peters S.A., Snoeys J., Upreti V.V., Zheng M., Hall S.D. (2015). Physiologically based pharmacokinetic modeling in drug discovery and development: A pharmaceutical industry perspective. Clin. Pharmacol. Ther..

[29] Shebley M., Sandhu P., Riedmaier A.E., Jamei M., Narayanan R., Patel A., Peters S.A., Reddy V.P., Zheng M., de Zwart L. (2018). Physiologically Based Pharmacokinetic Model Qualification and Reporting Procedures for Regulatory Submissions: A Consortium Perspective. Clin. Pharmacol. Ther..

[30] Berry S.J., Coffey D.S., Walsh P.C., Ewing L.L. (1984). The development of human benign prostatic hyperplasia with age. J. Urol..

[31] Varma M., Morgan J.M. (2010). The weight of the prostate gland is an excellent surrogate for gland volume. Histopathology.

[32] Koeppe M.O., Cristofoletti R., Fernandes E.F., Storpirtis S., Junginger H.E., Kopp S., Midha K.K., Shah V.P., Stavchansky S., Dressman J.B. (2011). Biowaiver monographs for immediate release solid oral dosage forms: Levofloxacin. J. Pharm. Sci..

[33] Hirano T., Yasuda S., Osaka Y., Kobayashi M., Itagaki S., Iseki K. (2006). Mechanism of the inhibitory effect of zwitterionic drugs (levofloxacin and grepafloxacin) on carnitine transporter (OCTN2) in Caco-2 cells. Biochim. Biophys. Acta..

[34] Mitsuboshi S., Yamada H., Nagai K., Kazuyuki U. (2016). No effect of protein binding ratio of levofloxacin in hemodialysis patients. Ren. Replace. Ther..

[35] Uchimura T., Kato M., Saito T., Kinoshita H. (2010). Prediction of human blood-to-plasma drug concentration ratio. Biopharm. Drug Dispos..

[36] Chien S.C., Rogge M.C., Gisclon L.G., Curtin C., Wong F., Natarajan J., Williams R.R., Fowler C.L., Cheung W.K., Chow A.T. (1997). Pharmacokinetic profile of levofloxacin following once-daily 500-milligram oral or intravenous doses. Antimicrob. Agents Chemother..

[37] Overholser B.R., Kays M.B., Lagvankar S., Goldman M., Mueller B.A., Sowinski K.M. (2005). Pharmacokinetics of intravenously administered levofloxacin in men and women. Pharmacotherapy.

[38] Hutschala D., Skhirtladze K., Zuckermann A., Wisser W., Jaksch P., Mayer-Helm B.X., Burgmann H., Wolner E., Müller M., Tschernko E.M. (2005). In vivo measurement of levofloxacin penetration into lung tissue after cardiac surgery. Antimicrob. Agents Chemother..

[39] Ambrose P.G., Grasela D.M., Grasela T.H., Passarell J., Mayer H.B., Pierce P.F. (2001). Pharmacodynamics of fluoroquinolones against Streptococcus pneumoniae in patients with community-acquired respiratory tract infections. Antimicrob. Agents Chemother..

[40] Andes D., Anon J., Jacobs M.R., Craig W.A. (2004). Application of pharmacokinetics and pharmacodynamics to antimicrobial therapy of respiratory tract infections. Clin. Lab. Med..

[41] Dalhoff A., Schmitz F.J. (2003). In vitro antibacterial activity and pharmacodynamics of new quinolones. Eur. J. Clin. Microbiol. Infect. Dis..

[42] Tasso L., de Andrade C., Dalla Costa T. (2011). Pharmacokinetic/pharmacodynamic modelling of the bactericidal activity of free lung concentrations of levofloxacin and gatifloxacin against Streptococcus pneumoniae. Int. J. Antimicrob. Agents.

[43] (2026). Performance Standards for Antimicrobial Susceptibility Testing.

[44] Marchand S., Frasca D., Dahyot-Fizelier C., Breheret C., Mimoz O., Couet W. (2008). Lung microdialysis study of levofloxacin in rats following intravenous infusion at steady state. Antimicrob. Agents Chemother..

[45] Van der Deen M., de Vries E.G., Timens W., Scheper R.J., Timmer-Bosscha H., Postma D.S. (2005). ATP-binding cassette (ABC) transporters in normal and pathological lung. Respir. Res..

[46] Campbell L., Abulrob A.N., Kandalaft L.E., Plummer S., Hollins A.J., Gibbs A., Gumbleton M. (2003). Constitutive expression of p-glycoprotein in normal lung alveolar epithelium and functionality in primary alveolar epithelial cultures. J. Pharmacol. Exp. Ther..

[47] Matsuo Y., Yano I., Ito T., Hashimoto Y., Inui K. (1998). Transport of quinolone antibacterial drugs in a kidney epithelial cell line, LLC-PK1. J. Pharmacol. Exp. Ther..

[48] Xia B., Heimbach T., Lin T.H., He H., Wang Y., Tan E. (2012). Novel physiologically based pharmacokinetic modeling of patupilone for human pharmacokinetic predictions. Cancer Chemother. Pharmacol..

[49] Miller N.A., Reddy M.B., Heikkinen A.T., Lukacova V., Parrott N. (2019). Physiologically Based Pharmacokinetic Modelling for First-In-Human Predictions: An Updated Model Building Strategy Illustrated with Challenging Industry Case Studies. Clin. Pharmacokinet..

[50] Bellmann R., Kuchling G., Dehghanyar P., Zeitlinger M., Minar E., Mayer B.X., Müller M., Joukhadar C. (2004). Tissue pharmacokinetics of levofloxacin in human soft tissue infections. Br. J. Clin. Pharmacol..

[51] Zeitlinger M.A., Traunmüller F., Abrahim A., Müller M.R., Erdogan Z., Müller M., Joukhadar C. (2007). A pilot study testing whether concentrations of levofloxacin in interstitial space fluid of soft tissues may serve as a surrogate for predicting its pharmacokinetics in lung. Int. J. Antimicrob. Agents.

[52] Conte J.E., Golden J.A., McIver M., Zurlinden E. (2006). Intrapulmonary pharmacokinetics and pharmacodynamics of high-dose levofloxacin in healthy volunteer subjects. Int. J. Antimicrob. Agents.

[53] Daneman N., Lu H., Redelmeier D.A. (2015). Fluoroquinolones and collagen associated severe adverse events: A longitudinal cohort study. BMJ Open.

[54] Lee C.C., Lee M.G., Hsieh R., Porta L., Lee W.C., Lee S.H., Chang S.S. (2018). Oral Fluoroquinolone and the Risk of Aortic Dissection. J. Am. Coll. Cardiol..

[55] Paglia M., Peterson J., Fisher A.C., Qin Z., Nicholson S.C., Kahn J.B. (2010). Safety and efficacy of levofloxacin 750 mg for 2 weeks or 3 weeks compared with levofloxacin 500 mg for 4 weeks in treating chronic bacterial prostatitis. Curr. Med. Res. Opin..

[56] Naber K.G. (2008). Management of bacterial prostatitis: What’s new?. BJU Int..

[57] Weidner W., Schiefer H.G., Krauss H., Jantos C., Friedrich H.J., Altmannsberger M. (1991). Chronic prostatitis: A thorough search for etiologically involved microorganisms in 1461 patients. Infection.

[58] Lock G.A., Helfer V.E., Dias B.B., Torres B.G.S., De Araújo B.V., Dalla Costa T. (2023). Population pharmacokinetic modeling of the influence of chronic and acute biofilm-forming Pseudomonas aeruginosa lung infection on ciprofloxacin free pulmonary and epithelial lining fluid concentrations. Eur. J. Pharm. Sci..

[59] Madaras-Kelly K.J., Ostergaard B.E., Hovde L.B., Rotschafer J.C. (1996). Twenty-four-hour area under the concentration-time curve/MIC ratio as a generic predictor of fluoroquinolone antimicrobial effect by using three strains of Pseudomonas aeruginosa and an in vitro pharmacodynamic model. Antimicrob. Agents Chemother..

[60] Forrest A., Nix D.E., Ballow C.H., Goss T.F., Birmingham M.C., Schentag J.J. (1993). Pharmacodynamics of intravenous ciprofloxacin in seriously ill patients. Antimicrob. Agents Chemother..

[61] Benko R., Matuz M., Doro P., Peto Z., Molnar A., Hajdu E., Nagy E., Gardi J., Soos G. (2007). Pharmacokinetics and pharmacodynamics of levofloxacin in critically ill patients with ventilator-associated pneumonia. Int. J. Antimicrob. Agents.

[62] Öbrink-Hansen K., Hardlei T.F., Brock B., Jensen-Fangel S., Kragh Thomsen M., Petersen E., Kreilgaard M. (2015). Moxifloxacin pharmacokinetic profile and efficacy evaluation in empiric treatment of community-acquired pneumonia. Antimicrob. Agents Chemother..

[63] Cen Y., Shan Y., Zhao J., Xu X., Nie Z., Zhang J. (2023). Multiple drug transporters contribute to the brain transfer of levofloxacin. CNS Neurosci. Ther..

